# Rubik’s Cube as Reconfigurable Microfluidic Platform for Rapid Setup and Switching of Analytical Devices

**DOI:** 10.3390/mi13122054

**Published:** 2022-11-24

**Authors:** Xiaochen Lai, Yanfei Sun, Mingpeng Yang, Hao Wu

**Affiliations:** 1 School of Automation, Nanjing University of Information Science & Technology, Nanjing 210044, China; 2 Jiangsu Collaborative Innovation Center of Atmospheric Environment and Equipment Technology (CICAEET), Nanjing University of Information Science & Technology, Nanjing 210044, China; 3 State Key Laboratory of Precision Measuring Technology and Instruments, Tianjin University, Tianjin 300072, China

**Keywords:** modular microfluidics, reconfiguration, on-site deployment, pollutant testing, smart instrumentation

## Abstract

Microfluidics technology plays an important role in modern analytical instruments, while the modular design of microfluidics facilitates the reconfiguration of analytical instrument functions, making it possible to deploy on-demand systems in the field. However, modular design also faces the challenges such as connection reliability and reconfiguration convenience. Inspired by the self-locking structure of the Rubik’s cube, a modular, reconfigurable microfluidic instrument architecture is proposed in this paper. The system has a self-locking structure of Rubik’s cube components and an O-ring-based alignment and sealing mechanism, which enables reliable interconnection and rapid rearrangement of microfluidic modules by simply rotating the faces of the microfluidic cube. In addition, the system is capable of integrating a variety of customized modules to perform analysis tasks. A proof-of-concept application of detecting multiple pollutants in water is demonstrated to show the reconfigurable characteristics of the system. The findings of this paper provide a new idea for the design of microfluidic analytical instrument architectures.

## 1. Introduction

At present, analytical instruments play an important role in various fields, such as food safety, agriculture, animal husbandry, and public health. In addition to improving performance, trends in analytical instrumentation include miniaturization, automation, and portability. To achieve these goals, the utilization of microfluidics technology is indispensable. Microfluidics technology refers to the use of micron-scale fluid channels to manipulate the fluid in the volume of microliters or nanoliters. Microfluidics has many advantages over traditional laboratory methods, such as lowering sample and reagent consumption, reducing cross-contamination risk, saving manual operation, increasing throughput, and reducing reaction time. Based on these advantages, microfluidic technology has become an inevitable topic in modern analytical instruments involving capillary electrophoresis [1,2], gas/liquid chromatography [3,4], polymerase chain reactions (PCR) [5,6,7], flow cytometry [8,9], and molecular diagnostics [10,11,12]. During the COVID-19 pandemic, rapid testing methods and devices based on microfluidics technology were developed and used [13,14,15] to prevent the large-scale spread of the pandemic through timely diagnosis, avoid the adverse impact of the pandemic on economic and public health, and demonstrate the bright future of microfluidics in analytical instrumentation.

However, despite the promising potential for miniaturization and automation, regular microfluidic technologies also face some challenges. An important limitation is the lack of flexibility to deal with varying application scenarios due to the monolithic design of microfluidic chips. For the existing analysis devices based on microfluidics, their functions are often limited by the compact design and specific purpose of use. In such systems, if some test parameters need to be changed, it is often necessary to redesign the whole microfluidic system. However, for analytical instruments, the system function is not always the same but often needs to be adjusted according to the needs; for example, changing the channel size for varying droplet volume or changing the sensors used for different analytes. In this context, the invariable function of microfluidics fails to meet the diversified application scenarios. As a result, end users end up redesigning or modifying the system for customized applications, which increases the requirements for user expertise and escalates the development costs.

The emergence of the modular microfluidic system provides a new way to solve this problem [16,17]. By combining sensors, actuators, and fluid channels into freely reconfigurable systems, the functionality of microfluidic systems can be adjusted on demand for specific applications. For example, Owens et al. proposed a Lego-type modular microfluidic system based on a micro-milling process and demonstrated the rapid function-switching ability of the system for different applications [18]. Gimenez-Gomez et al. constructed a modular analysis system based on magnetic connections, which enabled the determination of various environmental variables [19] and pollutants [20] through functional modules that could be freely replaced and combined. Dekker et al. implemented a micro-laboratory that can be configured on demand using a variety of functional modules, such as chambers, sensors, and actuators [21], and demonstrated the application of its configuration as a Coulter counter [22] and a cell culture and liquid dosing platform [23].

However, compared with the monolithic microfluidic system, the modular system generally has inherent defects such as difficult alignment, poor pressure resistance, and fluid leakage due to the discrete module design. To address these problems, additional strengthening structures have to be added to the modular design, such as using dedicated connection modules and backplates [18,24], connection pins [25], clamps and screws [22,26], and magnets [27,28] to strengthen the connection between modules. In this paper, we propose a modular microfluidic analytical system based on an interconnecting strategy using the interlocking mechanism of the Rubik’s cube. Adapted from our previous research work [29], the proposed modular system enables rapid switching of functions through simple twisting. In this paper, we utilize the unique self-locking mechanism of the Rubik’s cube to enable the self-aligned and reliable connection between modules without the use of threaded fitting and other means to obtain rapid reconfiguration, achieving a trade-off between performance and convenience. Moreover, the modular microfluidic system could integrate a variety of custom modules, such as sensors and actuators, for complex analytical tasks. By combining different kinds of microfluidic modules through cube permutation, one can build on-demand analytical instrument systems. As a proof of concept, the analysis of multiple pollutants in water was performed by reconfiguring the system from a configuration of electrochemistry-based heavy metal sensing to another configuration of colorimetric nitrite ion detection. The analytical instrument architecture based on a modular design proposed in this paper realizes on-demand configuration and rapid switching of analytical functions and can be used as a general platform for the on-site deployment of customized analysis systems in resource-limited settings. In addition, the system also has the potential to expand to higher-order Rubik’s cubes and integrate more functional modules for more complex instrumentation tasks.

## 2. Materials and Methods

### 2.1. Design of Rubik’s Cube-like System

**Figure 1:**
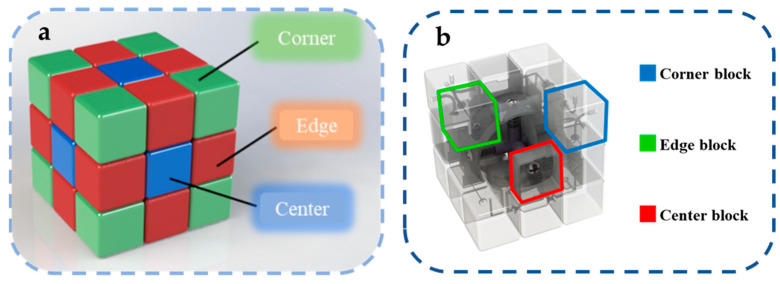
(**a**) The basic parts in a classic 3 × 3 × 3 Rubik’s cube, including corners, edges, and centers; (**b**) The illustration of the microfluidic cube presented in this paper.

According to the structural characteristics of the Rubik’s cube, the reconfigurable microfluidic system based on Rubik’s cube is shown in Figure 1. Similar to the classic 3 × 3 × 3 Rubik’s cube, the Rubik’s cube-like microfluidics mainly consists of several parts: modules on cube vertices (corner block), modules at the edge center (edge block), modules on face centers (center block), and Rubik’s cube core and fasteners. Among them, the corner blocks mainly serve for the fluid in/output, steering, and junctions, while the edge block is mainly responsible for specific fluid functions (transport, reaction, mixing, sensing, actuation, etc.). There are hollow microfluidic channels inside the corner blocks and the edge blocks, which can be connected with the adjacent modules and can be switched to different modules by the rotation of the cube layers.

Because the Rubik’s cube can be freely reorganized by rotation and can be operated without breaking the integrity of the parts, it is ideal for the design of reconfigurable microfluidic architectures: Firstly, the Rubik’s cube has a very compact mechanical structure [30], which can be applied to the tight integration of a large number of different functional modules. Secondly, a third-order Rubik’s cube has a total of 4.3 × 10^19^ possible arrangements. If each arrangement corresponds to a possible system configuration, then using the Rubik’s cube as a microfluidic system can achieve an almost infinite variety of microfluidic configurations. More importantly, it has been proved through mathematical methods that, for the third-order Rubik’s cube, the conversion between any two permutations can be completed only within 20 twists [31]. Therefore, the Rubik’s cube-like system has very good simplicity and ease of use, allowing the rapid transformation of microfluidic configurations.

### 2.2. Module Design

**Figure 2:**
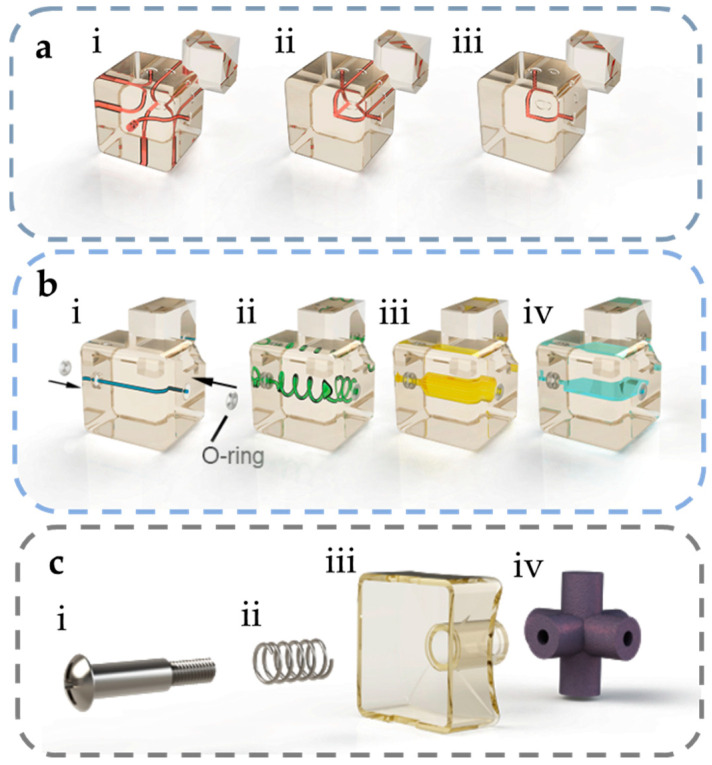
The design of the major components of the Rubik’s cube-like microfluidic cube: (**a**) the corner blocks, including in/outlet (**i**), turning (**ii**), and junction (**iii**) blocks; (**b**) the edge blocks, including straight (**i**) and spiral (**ii**) channels, 3D (**iii**), and planar (**iv**) chambers; (**c**) other parts of the cube, including the screw (**i**), spring (**ii**), center block (**iii**), and cube core (**iv**).

Corner blocks are functional modules located at the eight vertices of the Rubik’s cube. According to the structure of Rubik’s cube, a corner block is adjacent to three edge blocks, so the corner block is suitable for the modules that need to be interconnected with multiple modules, such as three-way junctions and steering channels. In addition, when a corner block is used as an inlet and outlet module, it can also be used as a 3-in-1 port to connect three fluid channels at the same time, avoiding the unnecessary occupation of the spaces that could be used for other functional modules. Figure 2 shows a schematic of the basic design of several corner blocks, including in/outlets, three-way junctions, and steering channels.

The edge blocks are located in the middle of the Rubik’s cube 12 edges, and each edge block is adjacent to two corner blocks. Therefore, the edge block as a microfluidic channel has only two ports, and the fluid flows in from one adjacent corner block and out to the other adjacent corner block. Having only two ports makes the edge very suitable to be integrated as a serial component in microfluidic systems because only the impact on a single branch should be considered when replacing an edge block, which facilitates the reconfiguration of microfluidic functions. Figure 2a,b shows several basic designs of the edge blocks, including straight channels, spiral channels, chambers, etc. The edge block can also integrate with other devices (sensors and actuators) for more versatile functions.

For center blocks on the center of each 6 faces, the design of microfluidic channels in these blocks is not considered in this paper. Although adding microfluidic structures to the center blocks and allowing them to interconnect with the edges can further increase the complexity of the system and achieve more functions through more possible combinations, it also causes some problems. Firstly, in the original design of the Rubik’s cube, to make the cube rotate smoothly, the thickness of the center blocks was much smaller than that of the edge block and corner block, so there was less room for the construction of microfluidic channels and other functional structures. Secondly, the center blocks need to be connected to the cube core with screws, so the fluid channel must bypass the through hole reserved for the screws, which sets limits on the fabrication technology and module functions. More importantly, there are 4 orientations for each center block. If the center blocks were used as functional modules, the orientation of the block must be considered during the configuration process, which complicates the reconfiguration of the microfluidic function. When considering the above factors, the center blocks of the Rubik’s cube were not used as other microfluidic functional modules here but only as the non-functional modules to fasten all the modules of the Rubik’s cube. Different from the edge and corner blocks, because there is no need for microfluidic channels inside, the central block of the face is designed as a hollow shell and connected with the cube core by screws, as shown in Figure 2. For all edge, corner, and center blocks, the length of the side of the block is 21.5 mm, as depicted in Figure S1.

**Figure 3:**
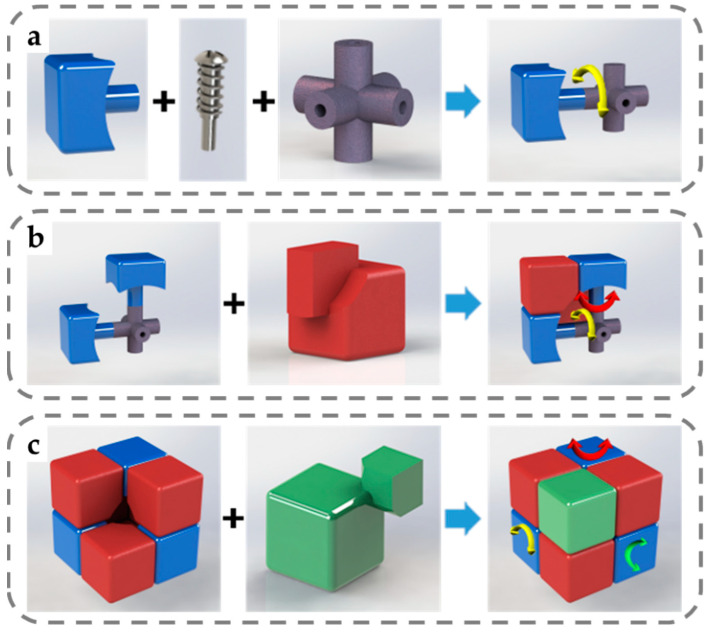
Illustration of how different blocks are interlocked with each other: (**a**) connecting center blocks to the cube core with screws and springs; (**b**) the interlocking of 2 center blocks and an edge block, allowing edge blocks to rotate around one of the adjacent center blocks; (**c**) the interlocking of 3 edge blocks and a corner block, allowing rotation around any one of the 3 axes.

**Figure 4:**
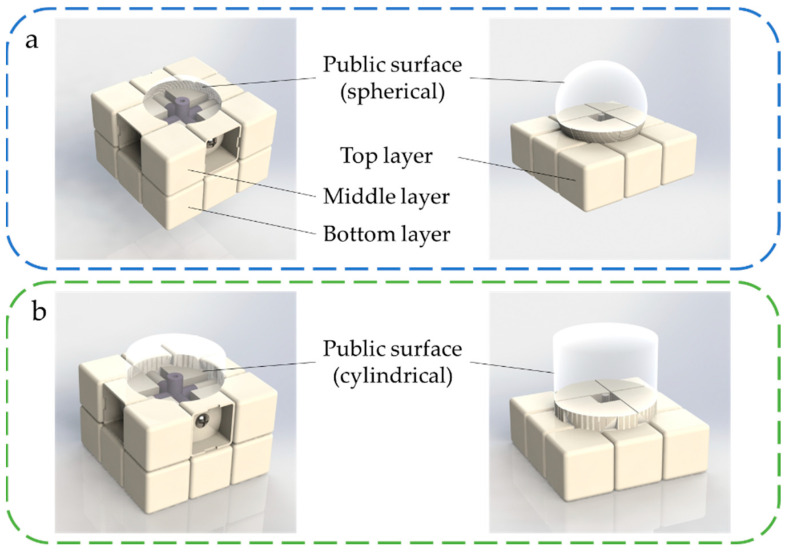
The interlocking of the top layer and the rest of the Rubik’s cube when the top layer rotates: (**a**) the spherical surface is used as the public surface; (**b**) the cylindrical surface is used as the common surface.

It should be noted that the Rubik’s cube modules are not simple cubes but three-dimensional shapes with additional protrusions and dents for interlock, as depicted in Figure 3. Taking the top layer of the rotating Rubik’s cube as an example, the protrusions on all the parts in the top layer can be embedded in the depression on the middle layer, and such interlock of the public surfaces ensures that the top layer of the whole module can only rotate around a single axis, as shown in Figure 4a. Therefore, the parameter setting of the public curvature on the edge block, corner block, and center block is the key to achieving smooth rotation and maintaining the unity of the Rubik’s cube. When the radius of public curvature is too large, the center block of the cube will become thinner, and the resulting deformation will easily cause the Rubik’s cube to fall apart in the process of rotation. When the radius of curvature is too small, the connection between the protruding structure and the edge/corner blocks will be weak, which makes them prone to deformation and fracture.

After referring to the structure of several commercial Rubik’s cube toys, this paper designed and tested the geometric parameters of the interlocking structure of the Rubik’s cube parts. Different from Rubik’s classic cube design that uses a spherical surface as a public surface (Figure 4a), this paper used a modified design that uses three cylindrical surfaces instead, which is also commonly used in present Rubik’s cube toys, as shown in Figure 4b. The advantage of this design is that, on the one hand, it can strengthen the connection between the protrusion on the block and the block itself, therefore enhancing the interlock between adjacent modules without thinning the center blocks, to prevent the deformation and damage to the module in during the rotations. On the other hand, the cylindrical surface makes the adjacent modules have a larger contact area when the Rubik’s cube rotates, which provides a smoother switching between configurations.

### 2.3. Module Alignment and Leak Proofing

Alignment and sealing strategies are essential to modular microfluidic systems. A poor connection may lead to many problems, including the system’s pressure intolerance and the consequent risk of fluid leakage. Sealing of the microfluidic environment is also an important issue in Rubik’s cube-like system. Ideally, each microfluidic module in the Rubik’s cube has a smooth surface, which can be completely fitted to the adjacent modules under the pushing force from the center block; thus, fluid leakage should not occur. However, due to the limitation of manufacturing technology, the actual fabricated Rubik’s cube parts are bound to have a certain degree of surface roughness, so the adjacent surfaces may not fit completely after rotation, resulting in liquid leakage from the gap of adjacent modules. In addition to leak proofing, how to ensure the alignment of microfluidic channels between adjacent modules after the Rubik’s cube rotation is also a problem to be solved.

**Figure 5:**
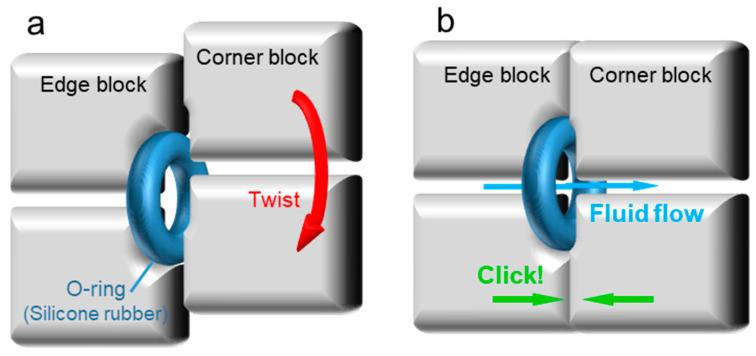
O-ring aided alignment and sealing strategy of the microfluidic modules: (**a**) when the Rubik’s cube is not rotated to the correct position, there will be a gap between the corner block and the edge block, and the phenomenon of fluid leakage will occur; (**b**) when the Rubik’s cube is rotated to the alignment position, the O-ring in the edge block will be embedded in the groove on the corner block to ensure the automatic alignment between the two modules and prevent leakage.

In order to deal with these problems, a microfluidic module alignment and sealing strategy based on silicone O-ring is proposed. Since the liquid leakage comes from the gap caused by the non-conformal contact between rigid modules, flexible materials are considered to be added between microfluidic modules to achieve complete fitting through the deformation of materials. Figure 5 shows the O-ring sealing strategy. On the edge block of the Rubik’s cube, annular grooves are designed around the entrance and exit of the microfluidic channel, which can be used to embed the silicone rubber O-ring. A small part of the silicone O-ring is exposed after embedding. There is also an annular groove on the corner block of the Rubik’s cube, whose contour is also the same as the O-ring, but the depth is shallow, as shown in Figure 5a. When the cube is rotated, the O-ring, which is embedded in the edge block, rotates with it and slides on the surface of the adjacent block. When the Rubik’s cube rotates completely and the microfluidic channel reaches the alignment position, the exposed part of the O-ring embedded in the edge block automatically fits into the groove on the corner block. At this time, the O-ring deforms under the spring thrust and fully fits the unsmooth surface on the edge block and corner block, thus avoiding leaving gaps between modules (Figure 5b). In addition, when rotating to the aligned position, the O-ring embedded in the corner block can increase the friction between the edge block and the corner block and prevent further relative displacement from achieving the automatic alignment between the edge block and the corner block.

### 2.4. Regular Modules Fabrication

The processing of microfluidic modules is divided into the following steps: printing, cleaning, and post-processing. Firstly, the cube modules were 3D printed using an SLA printer (Form2, Formlabs, Somerville, MA, USA) with a layer thickness of 0.1 mm, and transparent resin (FLGPCL04, Formlabs, Somerville, MA, USA) was used for observation from outside and optical applications. Support structure should not appear inside the modules. After printing, the support structures were removed from the printed modules, then immediately put into a container containing isopropanol (Rionlon Bohua (Tianjin) Pharmaceutical & Chemical Co., Ltd, Tianjin, China), and isopropanol was injected into the connectors of edge and corner blocks through a syringe to rinse out the remaining uncured photosensitive resin in the channel. Next, the printed parts in the isopropanol container were placed in the ultrasonic cleaning tank, and the water-separated ultrasonic treatment was performed for 10 min. Finally, all parts were removed from the container and left to dry at room temperature.

### 2.5. Custom Module Fabrication

**Figure 6:**
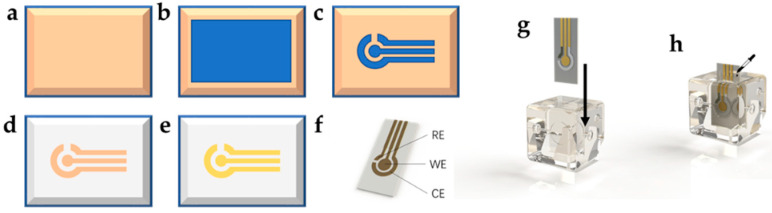
(**a**–**f**) Fabrication of three-electrode electrochemical sensor: the DBC ceramic substrate (**a**) laminated by a layer of photosensitive film (**b**); (**c**) the patterned photosensitive film is obtained after exposure and development; (**d**) copper is etched to obtain electrodes; (**e**) electroplating the electrode surface to obtain the gold three-electrode sensor; (**f**) illustration of the WE, CE, and RE of the three-electrode electrochemical sensor; (**g**,**h**) assembling the electrochemical sensor module; (**g**) inserting the electrochemical sensor into the edge block frame; (**h**) applying epoxy to the gap between the electrochemical sensor and the edge block for sealing.

In addition to regular modules, custom modules with integrations of other devices, such as sensors and actuators, were also fabricated. For example, the electrochemical sensor module processing method adopts the fabrication strategy of combining the 3D-printed microfluidic module frame with an independent electrochemical sensor. The electrochemical sensor is created by the etching process of direct bonding copper (DBC) ceramic substrate. The method is shown in Figure 6a–f: firstly, the photosensitive film is laminated to the copper-bonded Al_2_O_3_ ceramic; then, the photosensitive film is exposed to UV light under the cover of the film mask and then developed to obtain the patterned photosensitive film. Afterward, the copper is etched, and the photosensitive film is stripped to obtain the copper electrodes on the ceramic substrate. Finally, the electrochemical sensor is obtained by electroplating gold on the surface of the copper electrodes. After these steps, the electrochemical sensor can be further functionalized by surface treatment of each electrode according to the specific application. The dimensions for the electrochemical sensor are depicted in Figure S2.

The design of the microfluidic frame is shown in Figure 6g. In addition to the ordinary edge block, it has a rectangular structure, where the aforementioned electrochemical sensor can be inserted into the frame to connect with the fluid channel. In the sensing area of the electrochemical sensor (the circular area where WE, CE, and RE are located), a 500 μm thick storage chamber is also reserved to accommodate the electrochemical reactions, with a capacity of about 25 microliters. After the electrochemical sensor is inserted into the edge frame, the gap between the sensor and the frame is sealed with epoxy resin to prevent liquid leakage. Figure 6h shows the assembled electrochemical sensor module.

**Figure 7:**
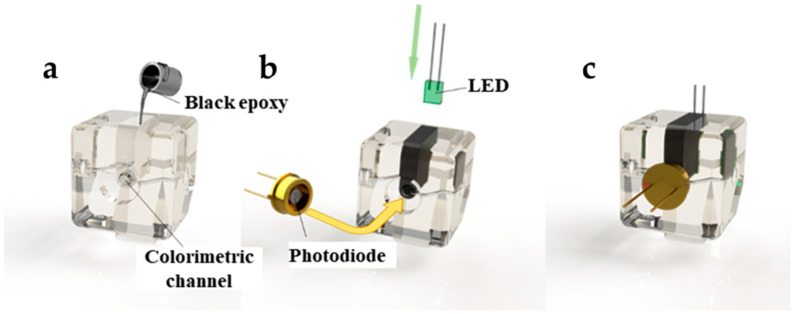
Colorimetric sensing module fabrication and assembly: (**a**) cast black epoxy resin at the periphery of the colorimetric channel in the 3D-printed colorimetric module; (**b**) assembly of the LED and silicon photodiode into the module; (**c**) the colorimetric sensing block after assembly.

A colorimetric sensing block is also presented here. The 3D-printed colorimetric sensing module frame, shown in Figure 7, was also adapted from the basic structure of the edge block. Similar to other microfluidic colorimeter designs, it has a cylindrical colorimetric channel with a cross-section radius of 750 μm and a length of 12 mm, whose direction is perpendicular to the outward surface of the edge block, and its two ends are connected to the two ports of the edge block. The total volume of the colorimetric channel is only 21 μL, which is much smaller than the volume of mL in traditional cuvettes. Therefore, only a small amount of samples and reagents are needed to complete the detection, which significantly reduces the consumption of samples and reagents. A cavity is reserved at the periphery of the colorimetric channel, and black epoxy resin can be poured into the cavity after 3D printing to block the ambient light from the colorimetric channel. In addition, the colorimetric module has a reserved space inside, where monochromatic light–emitting diode (LED) can be inserted into the module, and the emitted light can pass through the colorimetric path in the outside direction. A concave is also reserved on the outside of the block, opposite to the LED, where a photodetector (e.g., a silicon photodiode) can be embedded. In this module, the silicon photodiode is packaged in TO-5, and its circular profile can be firmly embedded in the colorimetric module by interference fit and aligned with the optical path to receive the light emitted by the LED and pass through the colorimetric channel. The shape and dimensions of the microfluidic channels in the colorimetric modules are depicted in Supplementary Figure S3.

## 3. Results and Discussion

### 3.1. System Assembly and Reconfiguration

This section discusses the characteristics of the Rubik’s cube and the assembly of the modular microfluidic system. Unless otherwise specified, the Rubik’s cube generally refers to the third-order Rubik’s cube, which is a cube with 27 main parts (3 × 3 × 3). There are a few tasks to be performed before the assembly and use of the Rubik’s cube-like system. Due to the characteristics of SLA technology forming layer by layer, the surface of the Rubik’s cube parts after 3D printing is not completely smooth, and the internal microfluidic channel cannot be observed from the outside. To facilitate later optical application, it is necessary to polish the outer surface of the edge block and corner block. Therefore, this paper used sandpaper to polish the surface of Rubik’s cube parts. The grit of sandpaper is gradually increased during the sanding process (from 1000 to 7000) until the microfluidic module becomes transparent and the internal structure can be clearly observed. The polished microfluidic module was then cleaned with deionized water. After drying, the outer surfaces of each module were sprayed with transparent varnish (Aerosol Paint, Sanvo Fine Chemicals, Guangzhou, China) and then left for 6 h until the varnish was completely cured before assembly.

**Figure 8:**
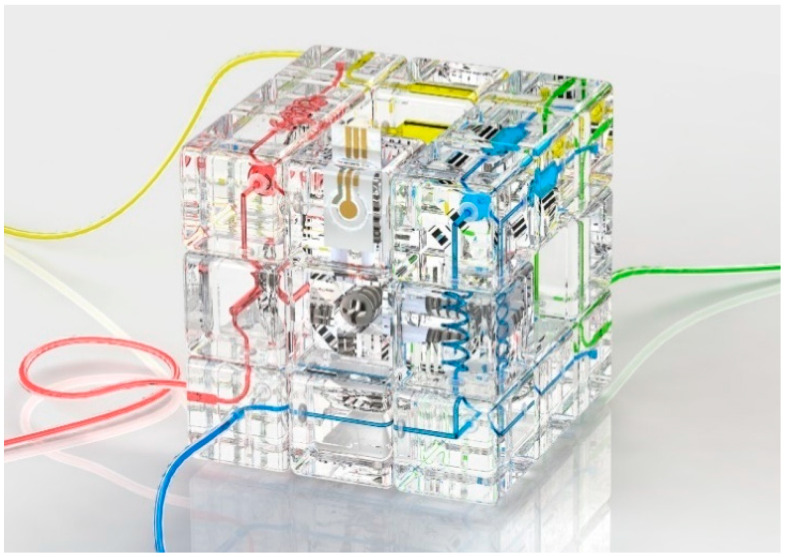
Illustration of the microfluidic cube integrated with the custom sensing modules.

Before assembling the microfluidic cube, silicone O-rings should be embedded in the two concave surfaces of all 12 edges, and then M3 threads should be drilled into the 6 holes of the cube core by manual thread tapping. Then the microfluidic cube should be assembled in the same way as the ordinary Rubik’s cube: First, five of the center blocks of the Rubik’s cube were connected to the central rotating shaft through the screws of M3 × 20 mm, and the springs of 0.6 mm (wire diameter) × 6 mm (helix diameter) × 15 mm (length). Then, the edge blocks and corner blocks of the Rubik’s cube were added to the assembly layer by layer. Finally, the remaining center block was connected to the rotating shaft of the Rubik’s cube. The tightness of the screws was adjusted so that each face of the cube rotated with moderate resistance. At this point, the microfluidic cube assembly was complete, as illustrated in Figure 8. We tested the pressure resistance of the microfluidic cube by blocking the outlet of the configuration and gradually increasing the inlet pressure. We tested the leakproofness of the system. Since the pressure resistance is tunable by adjusting the screws, the screws are tightened to a medium level, approximately four cycles of rotation (which is 2 mm into the cube core), and the setup for the test is shown in Figure S4. No leakage is observed in the process when inlet pressure is increased to 5.0 bar, demonstrating that the leakproofness of the assembly is sufficient for most microfluidic applications.

### 3.2. Electrochemical Module Tests

**Figure 9:**
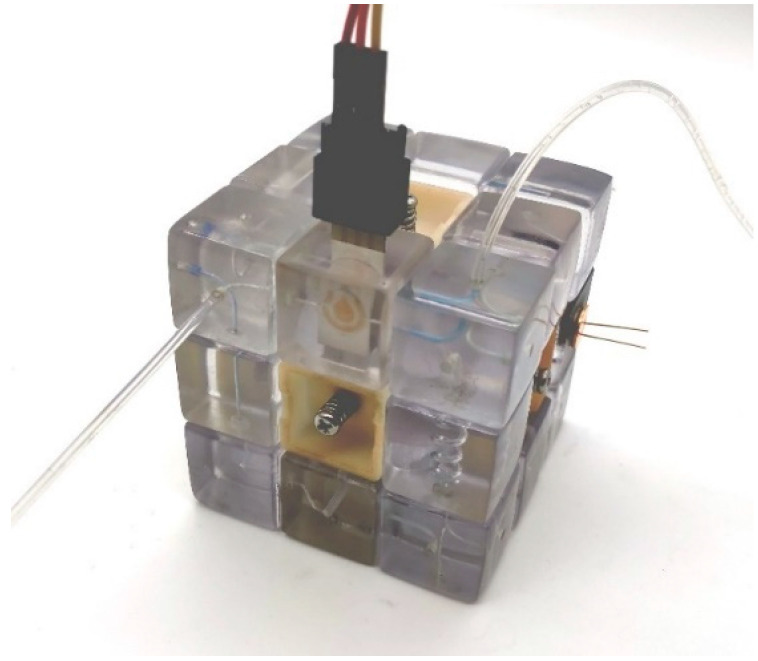
Experimental configuration of the electrochemical sensor module test.

When incorporated with electrochemical sensor modules, the microfluidic cube is capable of performing electrochemical analysis. Here, the electrochemical sensor module was validated by demonstrating the use of cyclic voltammetry and timed amperometry to test different potassium ferricyanide (K_3_[Fe(CN)_6_]) solutions with gradient concentrations. The experimental configuration is shown in Figure 9: assemble the electrochemical detection module completed above into the microfluidic cube, turn the input/outlet block to both ends of the electrochemical detection module, and then infuse the K_3_[Fe(CN)_6_] solution into the cube to fill the electrochemical module. Afterward, the pumping was stopped, and tests were carried out using the 3-electrode electrochemical sensor. It should be noted that this test is primarily a proof of concept demonstration of electrochemical module integration. The performance of the sensor is limited by fabrication and modification techniques, and there is still room for improvement.

**Figure 10:**
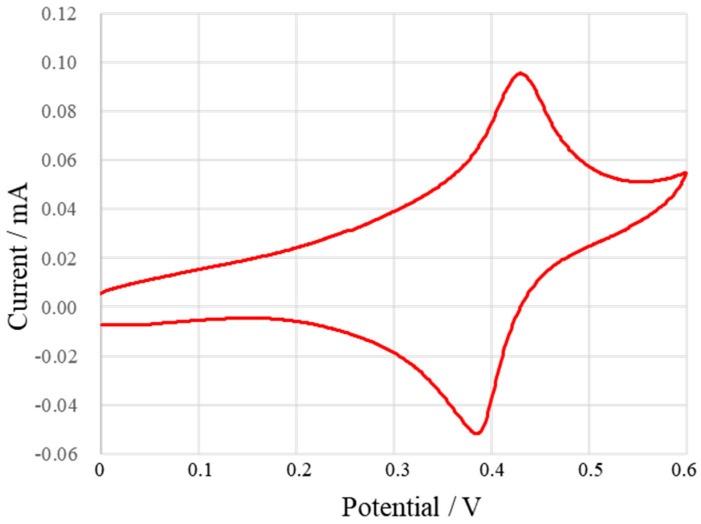
Cyclic voltammetry curve of (K_3_[Fe(CN)_6_]) solution.

Firstly, the cyclic voltammetry curve of 0.1 mmol/L (K_3_[Fe(CN)_6_]) solution was tested. The scanning interval was 0–0.6 V, and the scanning rate was 100 mV/s. Figure 10 shows the cyclic voltammetry curve of (K_3_[Fe(CN)_6_]). It can be observed that the oxidation peak appears at 0.430 V and the reduction peak appears at 0.386 V. The difference between the two seals is 0.044 V. The peak currents were 0.048 mA and 0.056 mA, respectively, and the ratio of peak currents was 85.7%.

**Figure 11:**
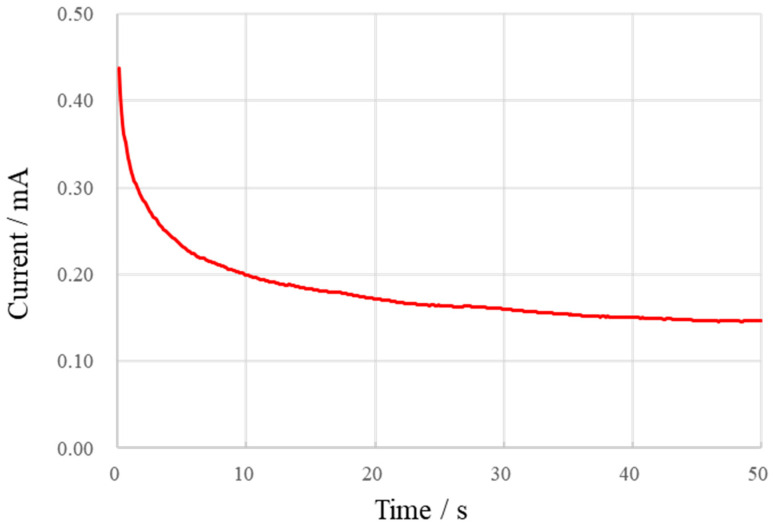
Chronoamperometry current curve of K_3_[Fe(CN)_6_] solution.

Then, the K_3_[Fe(CN)_6_] solution was tested using the chronoamperometry method. The I-t curve was recorded at an initial voltage of 0.43 V for the (K_3_[Fe(CN)_6_]) solution with a series of concentrations ranging from 0.1 mmol/L to 1 mmol/L. Figure 11 shows the I-t curve of 1 mmol/L. It is observed that the current tends to stabilize after 50 s, so the current at 50 s is taken as the steady-state current.

**Figure 12:**
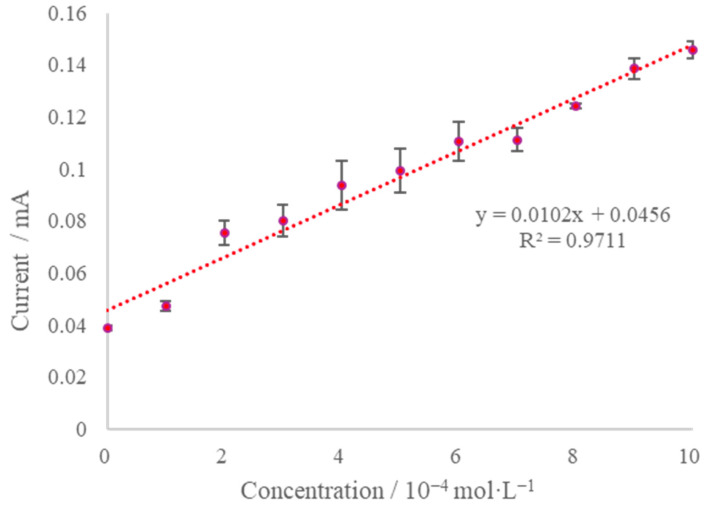
Steady-state current as a function of K_3_[Fe(CN)_6_] concentration in solution. The error bar represents the standard deviation of 3 measurements.

Figure 12 shows the steady-state current variation with different concentrations of K_3_[Fe(CN)_6_] solutions. As can be seen from the figure, the sensor exhibits reasonable linearity and sensitivity for timed current detection of K_3_[Fe(CN)_6_] and is expected to be usable for microfluidic detection applications with appropriate modifications.

### 3.3. Colorimetric Module Tests

The basic principle of the colorimeter is based on the Beer–Lambert law, which describes the attenuation of light as it propagates through a medium and explains how absorbance can be used to analyze the concentration of the analyte to be measured in a solution. The Beer–Lambert law states that the absorbance of a solution is proportional to the length of the light path through the solution and the concentration of the analyte to be measured. Therefore, after measuring the light intensity passing through the solution and calculating the absorbance, the concentration of the solution to be tested can be derived by the Beer–Lambert law.

**Figure 13:**
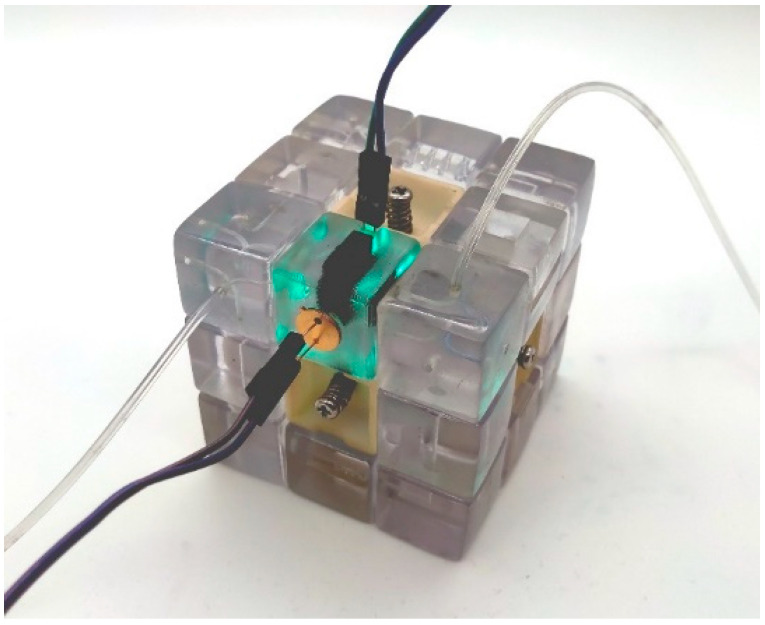
Experimental configuration of the colorimetric light sensor module.

Here we demonstrate the use of the microfluidic cube and colorimetric light sensor module to test glucose concentration in samples. In this application, the absorption peak of the colorimetric product is around 505 nm, so using a cyan LED with a central wavelength of 495–500 nm as the light source can achieve high sensitivity and reduce the influence of irrelevant components on the results. Silicon photodiode BPW21R (Vishay Semiconductor, Malvern, PA, USA) can be used, which has a high sensitivity at this wavelength. The microfluidic cube configuration in this test is shown in Figure 13: turn the inlet/outlet module to both ends of the colorimetric sensing module and then pass the solution to be tested into the module for testing.

The colorimetric reagent used was the GOD-PAP glucose assay kit (BioSino Bio-Technology and Science Inc., Beijing, China). According to the instructions of the kit, after mixing the colorimetric reagent, 1.5 mL of the colorimetric reagent was transferred into eleven 1.5 mL centrifuge tubes, and then 10μL of glucose solution was transferred into each centrifuge tube, with concentrations of 0, 30, 60, …, 300 mg/dL. The reagent in the centrifuge tube was mixed and left to stand at 37 °C for 15 min. Then, the reagents in the centrifuge tube were successively injected into the microfluidic cube at an interval of about 60 s for testing.

**Figure 14:**
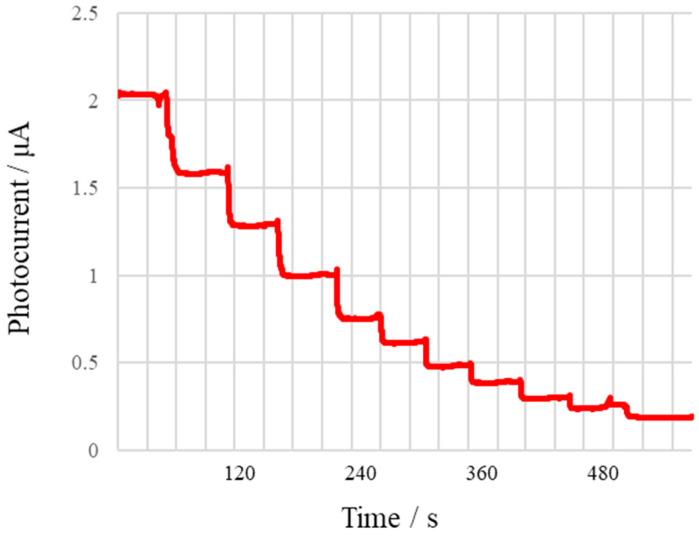
The curve of photocurrent as a function of time when the colorimetric mixture is injected into the module with an increasing concentration of glucose.

Figure 14 shows the curve of photocurrent change with time. The photocurrent is the current generated by the photoelectric effect in the light-receiving photocells and is proportional to the light intensity received by the detector. It can be observed that after infusion of a higher concentration of glucose solution, the photocurrent value is significantly attenuated, indicating that with the rise of glucose concentration in the sample, the light absorption of the solution increases continuously.

**Figure 15:**
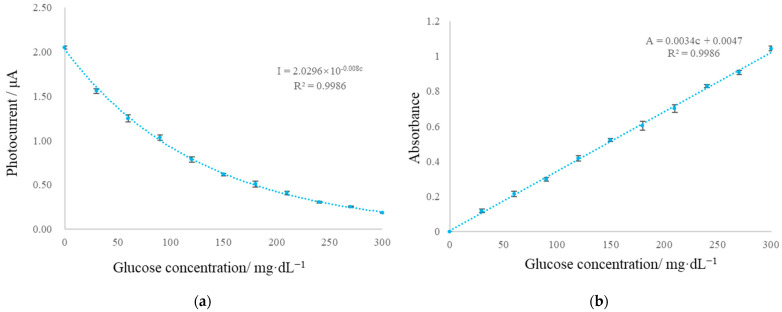
(**a**) Photocurrent and (**b**) calculated absorbance as functions of glucose concentration. The error bar represents the standard deviation of 3 measurements.

Figure 15a shows the relationship between photocurrent and solution concentration. The curve fitting results show that photocurrent intensity decays exponentially with the linear increase in glucose concentration in solution. The photocurrent generated by the light of the solution with glucose concentration 0 was taken as the photocurrent corresponding to the initial light intensity, and the absorbance of each solution was calculated according to the Beer–Lambert law, as shown in Figure 15b. The results show that the colorimetric sensor module has very good linearity and sensitivity and can meet the requirements of general colorimetric measurement by selecting appropriate light sources and detectors on demand.

### 3.4. Multiplexed Testing of Water Pollutants through Reconfiguration

Compared with the microfluidic system in other forms, the module rotation and reconstruction in the Rubik’s cube-like system can bring convenience to the rapid reconstruction of the microfluidic system, such as reorganizing the modules in a few twists. Here, this section demonstrates a rapid-switching water quality analyzing system based on the cube system, in which the system functions and the analyte to be measured can be switched by simply turning the Rubik’s cube.

Generally, water quality analysis equipment analyzes water pollutants based on a single principle. For example, a colorimeter is usually used for the colorimetric detection of nitrogen and phosphorus pollutants, while for the detection of metal ions, an ion analyzer based on electrochemistry is often used. For application scenarios that need to quantify multiple water pollutants, it is necessary to use a variety of analytical methods, which increases the experimental cost and cause issues such as poor portability and frequent sampling. Intelligent analytical instruments based on the microfluidic cube can give full play to the characteristics of changeable instrument architecture and can be configured as a colorimeter, an electrochemical analyzer, etc., according to the change in requirements to meet the needs to detect various water pollutants through a single system.

**Figure 16:**
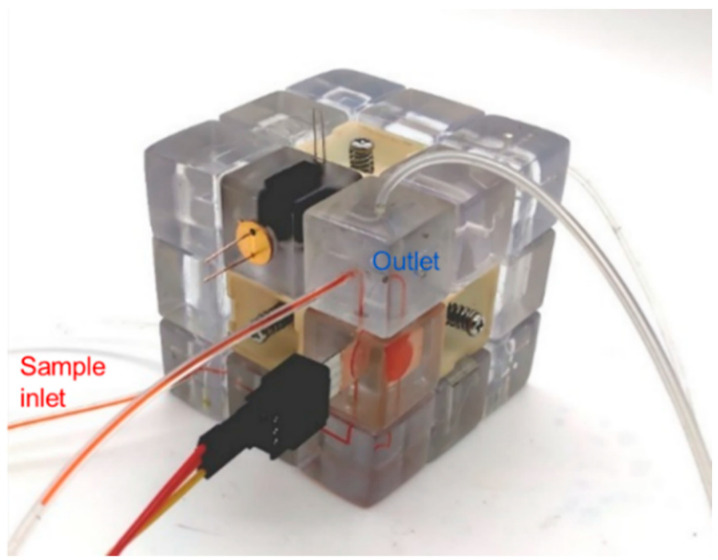
Configuration of the Rubik’s cube in its initial state (state 1).

**Figure 17:**
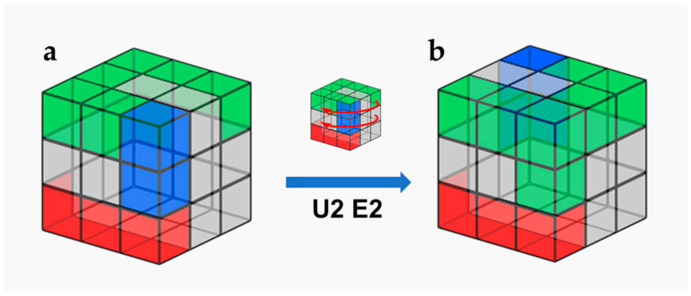
Schematic diagram of two configurations of the Rubik’s cube. (**a**) Initial configuration of the Rubik’s cube (state 1). (**b**) The reconfigured Rubik’s cube (state 2) after applying algorithm U2 E2. Red: modules shared by both configurations. Blue: The module used by configuration 1 alone. Green: The module used by configuration 2 alone.

**Figure 18:**
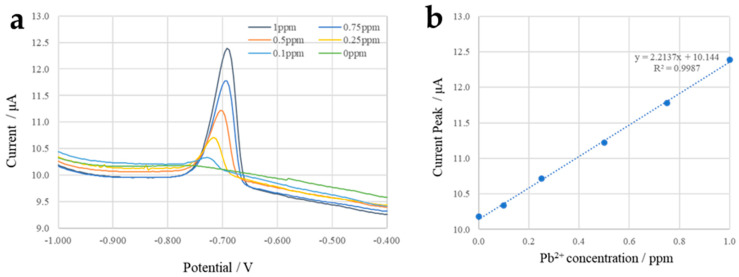
(**a**) Differential pulse anodic stripping voltammetry curves of different Pb^2+^ concentrations. (**b**) Peak current as a function of Pb^2+^ concentration.

The configuration of the Rubik’s cube-like system is shown in Figure 16: The initial state of Rubik’s cube (state 1) uses five modules: two in/outlet modules, one straight channel module, one steering module, and one electrochemical sensor module. These modules used in the initial state are shown in red and blue in Figure 17. In addition, some of the unused positions in state 1 are also configured with in/outlet modules, 3-way junction modules, spiral channel modules, colorimetric light sensor modules, etc. The positions of the unused blocks in the initial configuration are shown in green in Figure 18. The configuration of the initial state of the cube can be performed by applying Rubik’s cube algorithms in Singmaster Notation [32], which allow the intentional interchange of block positions. By rotating the top two layers of the Rubik’s cube 180° (the algorithm is U2 E2), as shown in Figure 18, the system can switch from the initial state to another state (state 2), and the function of the system can be switched from monitoring heavy metal ions in samples by electrochemical method to testing nitrite by colorimetric method.

To achieve the planned analysis, electrochemical sensor modules and colorimetric modules require some extra modification for specific detection: for the three-electrode electrochemical sensor, the reference electrode should be modified with Ag/AgCl to stabilize the reference electrode potential. Therefore, first, use electroplating to deposit silver on the reference electrode, then drop FeCl_3_ solution on the surface of the silver electrode to chlorinate the silver and achieve an AgCl coating. For the colorimetric detection of nitrite, the popular Griess test was used, where test reagents (Hubei Xinnongke Biotechnology Co. Ltd., Jingmen, China) and nitrite ion produce a magenta product with a peak absorbance of around 530 nm. In this case, the commonly used green LED with a center wavelength of 520–525 nm could be used for good sensitivity, and the detector could be the same photodiode BPW21R as the glucose colorimetric detector in Section 3.3. The system is intended for continuous and frequent sampling, so no rinsing strategy is employed between measurements.

The initial state configuration of Rubik’s cube can be used for continuous detection of heavy metal ions in water. When the wastewater containing electrolyte and heavy metal ions flows into the microfluidic cube, the concentration of heavy metal ions can be measured by differential pulse anodic stripping voltammetry using the electrochemical sensor module. Here, taking the detection of lead ions in water as an example, different concentrations of lead chloride (Pb^2+^ concentration: 0 ppm–1 ppm) in sodium acetate and acetic acid mixture buffer (0.1 mmol/L, pH = 5.2) simulating the wastewater sample were tested, and the tested solution was injected into the microfluidic cube at a flow rate of 50 μL/min. After filling the electrochemical sensor module, a constant potential of −1.0 V was applied to the WE. The heavy metal ions were deposited on the surface of the working electrode, and then differential pulse voltammetry was carried out on the electrochemical sensor to trace the content of Pb^2+^ according to the height of the dissolution peak. Table 1 shows the parameter settings of the differential pulse anodic stripping voltammetry for the electrochemical workstation (CHI-660E, CH Instruments, Austin, TX, USA) in this configuration.

Figure 18a shows differential pulse anodic stripping voltammetry curves of Pb^2+^ solutions with different concentrations, from which we learned that the dissolution peaks of Pb^2+^ are approximately −0.7 V. Figure 18b shows the curve of dissolution peak current as a function of concentration, indicating that the sensor has good linearity and sensitivity in the range from 0 ppm to 1 ppm, which can meet the needs for detecting lead ion released by ceramic hollowware according to FDA guidelines [33].

**Figure 19:**
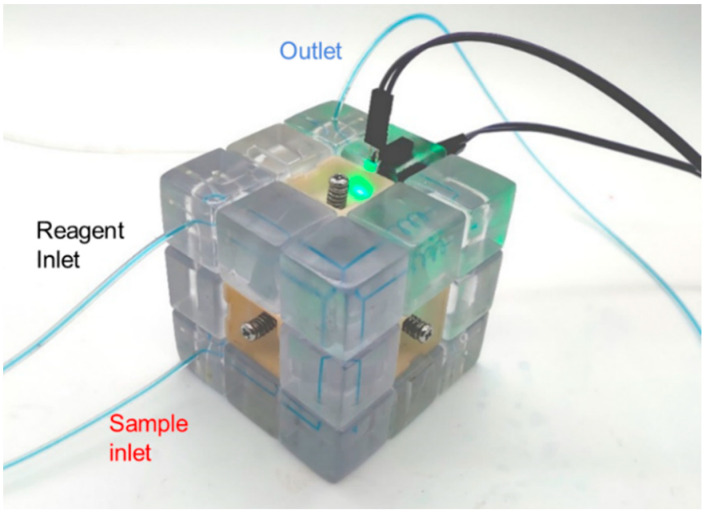
State of the Rubik’s cube after reconfiguration (state 2) used for the colorimetric detection of nitrite.

**Figure 20:**
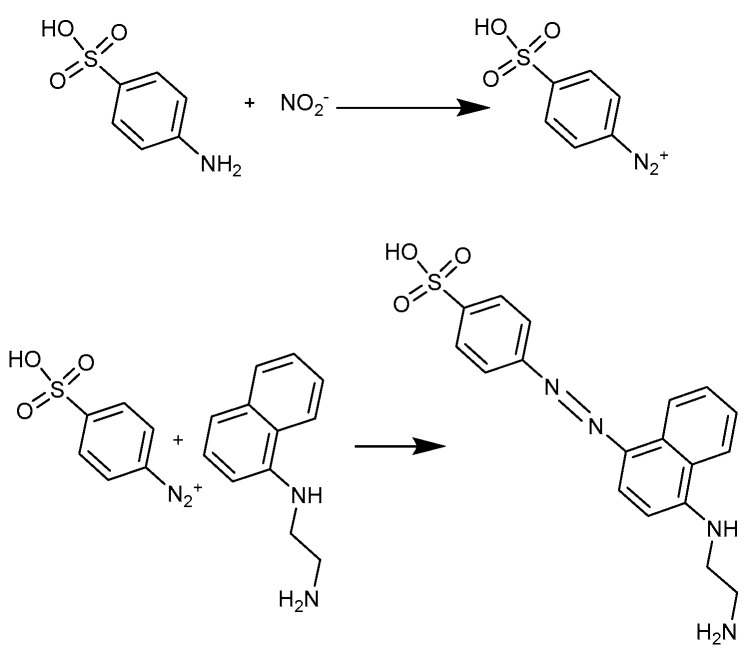
The Greiss test reactions.

When state 1 of the microfluidic cube is changed to state 2 by rotating the top two layers of the cube, the function of the instrument is switched from electrochemical heavy metal detection to colorimetric nitrite detection, as shown in Figure 19. Since state 2 and state 1 share the same inlet module, the sample inlet is unchanged, and the wastewater sample can enter the microfluidic cube from the same inlet, still at a flow rate of 50 μL/min. In this scene, we used sodium nitrite solution to simulate wastewater containing nitrite ions. At the same time, colorimetric reagents flow into the other inlet at the same rate. The sample meets the colorimetric reagent in the 3-way junction block and flows through the spiral channel block for mixing, and then flows into the colorimetric sensing module. After the mixed liquid flows out of the Rubik’s cube, the pumping is stopped, and the mixture is incubated for 15 min at room temperature. In the colorimetric module, the so-called Griess test took place (Figure 20).

The reaction produces a reddish-purple diazo dye in the presence of nitrite ions, so the color of the liquid can be used to determine the concentration of nitrite. The absorbance of each solution was calculated by taking the light intensity of the solution with a concentration of 0 as the initial light intensity.

**Figure 21:**
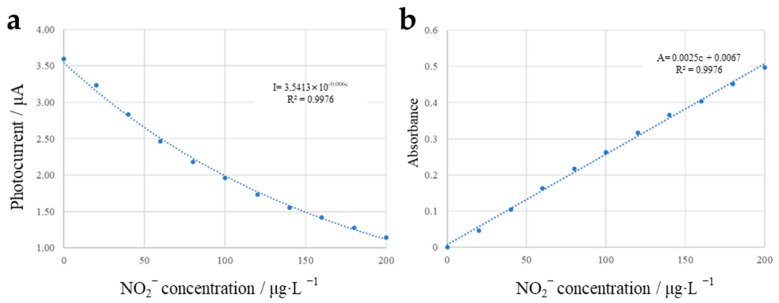
(**a**) Photocurrent and (**b**) absorbance as a function of nitrite ion concentration.

Figure 21a shows the photocurrent received by the silicon photodiode with the change in concentration of NO_2_^−^ in the sample. It can be inferred from the curve that the photocurrent decays exponentially with the increase in the concentration of NO_2_^−^. Figure 21b shows the correlation of the calculated absorbance with nitrite ion, and the two are linearly correlated with a sensitivity of 0.0025 AU/μg·L^−1^. The results show that the configuration could be used for the detection of nitrite ions with a detection range covering the common range of nitrite concentration in a variety of mediums, such as fermentation broth and aquaculture, and is suitable for the detection of nitrite in aquaculture and food processing applications [34].

## 4. Conclusions

In this paper, a Rubik’s cube microfluidic analysis device based on modular design is proposed. By integrating different sensing modules into the Rubik’s cube-like microfluidic system, different analysis systems can be configured and quickly switched. Through the multiplexed detection application of water pollution, the Rubik’s cube-like analysis system shows good analytical performance and convenience in reconfiguration, which is expected to be useful in field deployment and other customized microfluidic analysis scenarios. The system design utilizing Rubik’s cube may also inspire innovations in the architectures of analytical instruments. However, the proposed systems face challenges such as currently limited applicability in real scenarios due to the limited types of modules and requirements for prior knowledge to configure the cube. In the future, this research on this topic can be further extended in the following aspects:

(1) Electronic components used in the system can also be modularly designed (e.g., creating circuit contacts for adjacent blocks, similar to the connection of the microfluidic channels), and a unified power supply and data collection strategy for modules related to electronic components could be devised to improve the compactness and integrity of the system, enabling a more intelligent and automated analytical application.
(2) The microfluidic cube can be further extended to a higher-order Rubik’s cube structure (such as 4 × 4 × 4 or 5 × 5 × 5) to allow the integration of more functional modules in the system.
(3) The ease of use of the system is to be further improved to remove the barrier for end-users facilitating the proposed platform. Dedicated programs can be designed for the calculation of the algorithms that are used to configure the system to a specific state, simplifying the reconfiguration of the cube.
(4) For the construction of a more versatile analytical platform, a module library containing more functional modules should be built, and more types of different blocks, such as pumping/valving modules, heating/cooling modules, temperature sensing modules, active acoustic fluid mixing modules, fiber optic sensor modules, microscopic imaging modules, etc., will be very useful to be included.

## Figures and Tables

**Table 1:** Parameters of differential pulse stripping voltammetry setting for the detection of Pb^2+^.

Parameter/Unit	Value
Deposition potential/V	−1.0
Deposition time/s	120
Initial potential/V	−1.0
Final potential/V	−0.1
Potential increment/V	0.004
Pulse period/s	0.2
Pulse amplitude/V	0.05
Sampling width/s	0.02
Pulse width/s	0.06

## Data Availability

The experimental data are available from the authors.

## Supplementary Materials

The following supporting information can be downloaded at: https://www.mdpi.com/article/10.3390/mi13122054/s1. Figure S1: Dimensions of the major components of the microfluidic cube; Figure S2: Dimensions of the three-electrode electrochemical sensor; Figure S3: Dimensions of the colorimetric module.; Figure S4: Experimental setup of the pressure resistance test of the microfluidic cube.
