# Supplementary material for: Rubik’s Cube as Reconfigurable Microfluidic Platform for Rapid Setup and Switching of Analytical Devices
Source: Micromachines (Basel). 2022 Nov 24;13(12):2054. doi: 10.3390/mi13122054 (PMC9782984; doi:10.3390/mi13122054)
Supplement: Supplementary file 1 [file micromachines-13-02054-s001.zip › micromachines-2013338-supplementary.pdf]

Supplementary Information

Rubik's Cube as Reconfigurable Microfluidic Platform for Rapid Setup and Switching of Analytical Devices

Xiaochen Lai [1, 2, *], Yanfei Sun [1, 2], Mingpeng Yang [1, 2], Hao Wu[3]

1. School of Automation, Nanjing University of Information Science & Technology, Nanjing, 210044, China;
2. Jiangsu Collaborative Innovation Center of Atmospheric Environment and Equipment Technology (CI-CAEET), Nanjing University of Information Science & Technology, Nanjing, 210044, China
3. State Key Laboratory of Precision Measuring Technology and Instruments, Tianjin University, Tianjin 300072, China

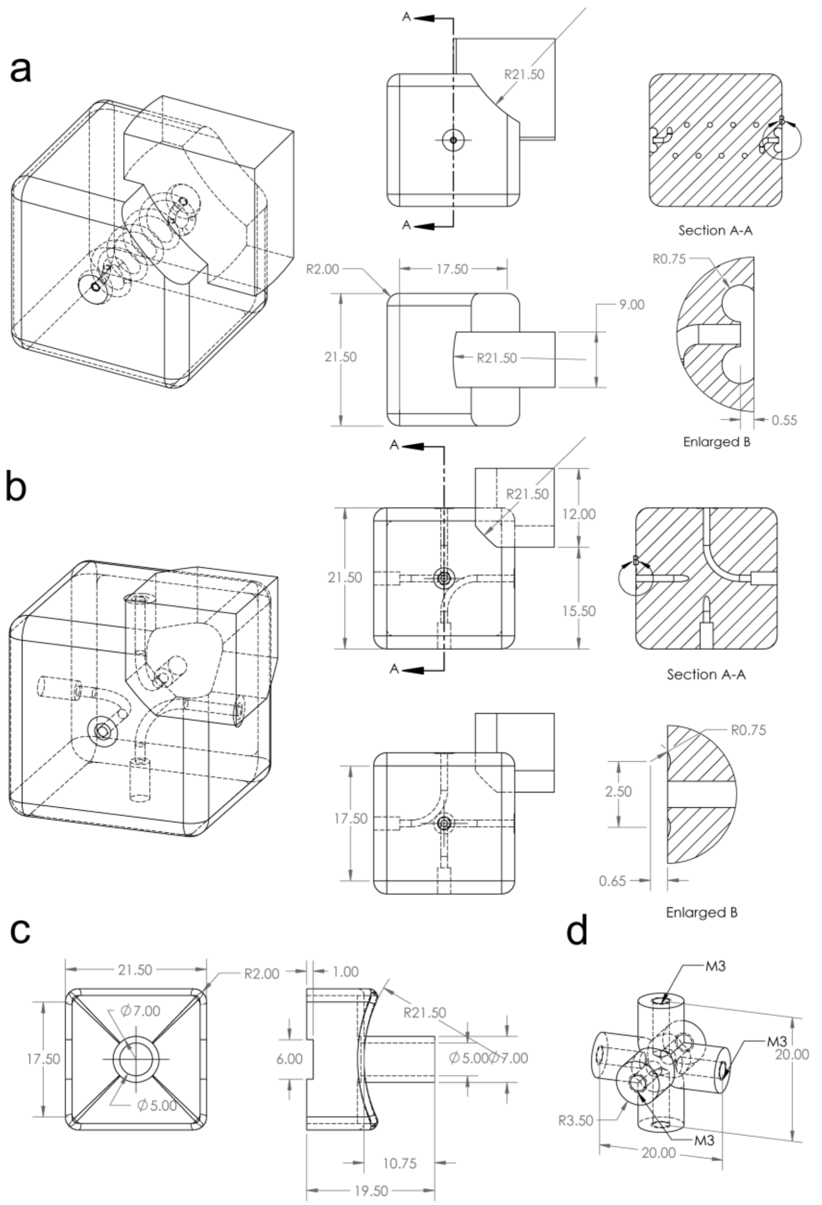

Figure S1 Dimensions of the major components of the microfluidic cube. (**a**). An edge block with a spiral channel. Right bottom: enlarged view of the O-ring embedding torus concave. (**b**). A corner block of 3-way inlets/outlets. Right bottom: enlarged view of the O-ring fitting torus concave. (**c**). A central block. (**d**).The cube core. All values are in mm.

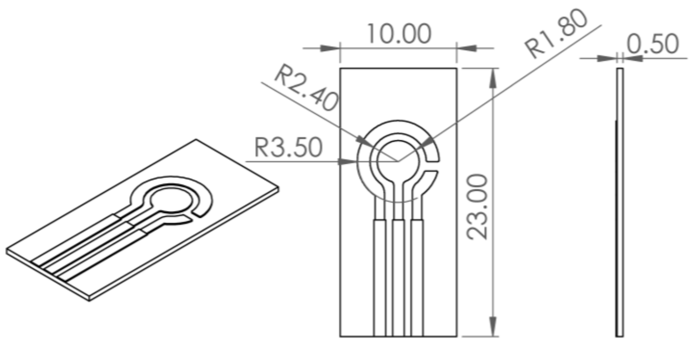

Figure S2. Dimensions of the three-electrode electrochemical sensor. Unit: mm.

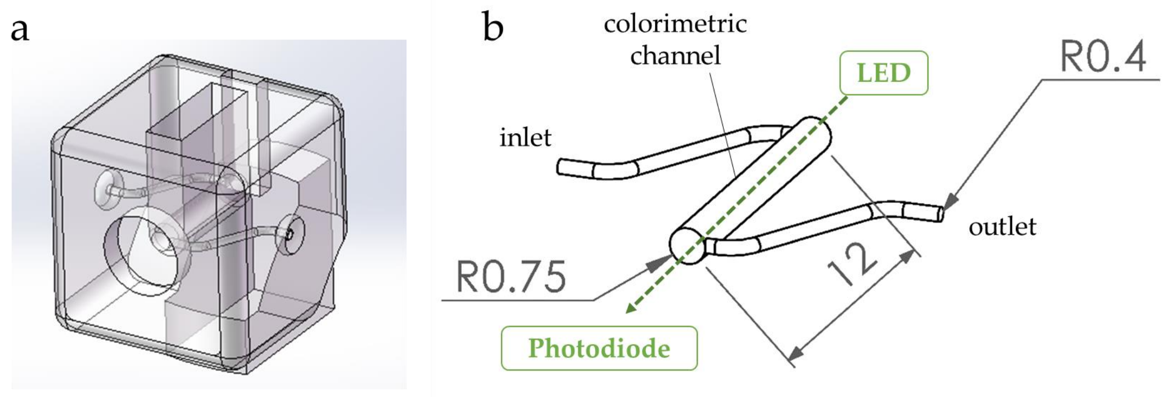

Figure S3. Dimensions of the colorimetric module. (**a**)**.** A transparent illustration of the colorimetric module frame (without black epoxy resin, LED, and photodiode). (**b**)**.** Geometry and dimensions of the actual fluid channel inside the module. Unit:mm.

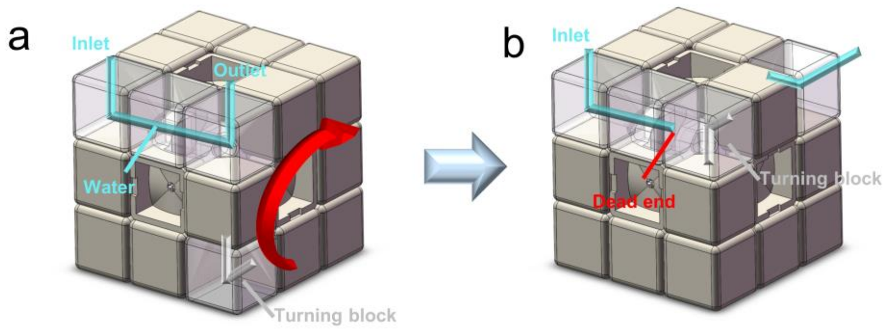

Figure S4. Experimental setup of the pressure resistance test of the microfluidic cube. (**a**)**.** At first, The microfluidic cube is configured to have 3 blocks: Inlet, straight channel and outlet. Water is injected into the cube to fill the channel. (**b**)**.** Afterward, turn a turning corner block to replace the outlet block to form a dead end for the microchannel. Then, the pressure generated by an air compressor and a pressure relief valve is applied to the inlet to verify the pressure resistance.
